# Comparison of area-length method by echocardiography versus full volume quantification by cardiac magnetic resonance imaging for the assessment of left atrial volume

**DOI:** 10.1186/1532-429X-14-S1-P297

**Published:** 2012-02-01

**Authors:** Peace Madueme, Kan N Hor, Joshua Germann, Wojciech Mazur, John L Jefferies, Michael Taylor

**Affiliations:** 1 Heart Institute, Cincinnati Children's Hospital Medical Center, Cincinnati, OH, USA; 2 Ohio Heart, Christ Hospital, Cincinnati, OH, USA

## Background

Left atrial (LA) size is a known predictor of adverse cardiovascular events. Echocardiography is the modality of choice for the evaluation of atrial size however cardiac magnetic resonance imaging (CMR) remains the gold standard for cardiac chamber volume quantification. We sought to compare atrial volume assessment using the biplane area-length method by echocardiography with full volume quantification by CMR.

## Methods

19 patients (mean age 20 ± 11years, 74% male) who had undergone CMR and echocardiography between June 2010 and June 2011 were retrospectively identified. The time interval between the two studies was 6 months or less. LA volume by echocardiogram was estimated using the biplane area-length method where: LA volume = (0.85 x Area4ch x Area2ch)/(Longest atrial length). The LA long axis dimension and area were measured in standard apical 2-chamber and apical 4-chamber views. Measured values were indexed to body surface area (BSA). CMR measurements were obtained from prospectively gated steady state free precession (SSFP) cine stack images obtained in a standard 4-chamber (horizontal long axis) plane. LA volumes were calculated using the Simpson’s method where: LA volume = LA area × (slice thickness + gap) for each slice. Slice thickness ranged from 5-7mm with contiguous slices of 5-7mm. The values were indexed to BSA. Statistics were summarized using measures of central tendency. Regression analysis and a Bland-Altman analysis were performed to assess the correlation and relationship between the two methods.

## Results

LA volumes estimated using the biplane area-length method by echocardiography were significantly less than the LA volumes estimated by full volume CMR quantification. The mean LA volume for echocardiography and CMR were 31.8 ± 10.8 and 43.8 ± 13.3 respectively (p = 0.007). The mean difference between LA volumes obtained by the two methods was 11.9 ± 11.7. Regression analysis demonstrated a linear correlation between the LA volumes estimated by echocardiography and by CMR (y = 12.6 + 0.4x, r = 0.54). This correlation was even stronger after the removal of two outliers. There were no significant differences between the ages, weights, heights or BSA in the patients between the two different studies.

## Conclusions

LA volume as measured by echocardiography using the biplane method consistently underestimated the LA volume when compared to CMR full volume quantification. Care should be taken in the clinical interpretation of LA volumes across these two different modalities.

## Funding

No internal or external funding was utilized for this study.

**Figure 1:**
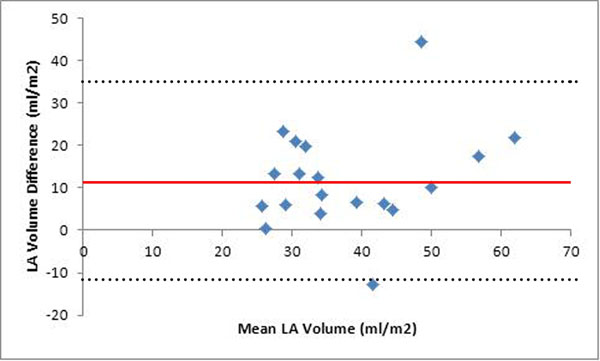
Bland-Altman plot for LA volume difference between echocardiography and CMR.

**Figure 2:**
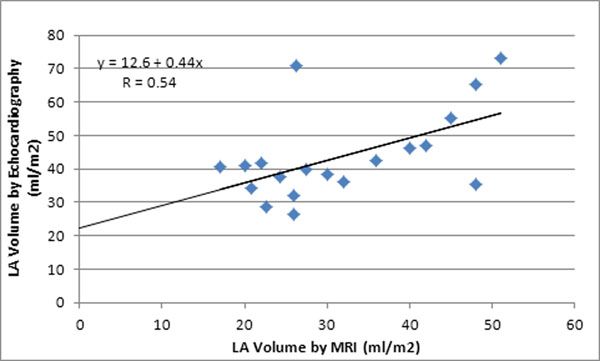
Correlation between LA volumes measured by echocardiography and CMR.

